# Supplementary material for: The genome sequence of the silver stretch spider, Tetragnatha montana (Simon, 1874) (Araneae: Tetragnathidae)
Source: Wellcome Open Res. 2024 May 22;9:288. [Version 1] doi: 10.12688/wellcomeopenres.21782.1 (PMC11863303; doi:10.12688/wellcomeopenres.21782.1)
Supplement: Peer review history

# Reviewer report: Reviewer response for version 1

**DOI:** 10.21956/wellcomeopenres.24059.r94604

**Published:** 2024-09-18

**Authors:** Feng Zhang

**Affiliations:** 1 Department of Entomology, Nanjing Agricultural University, Nanjing,, China

The authors report the availability of the genome sequence of *T. montana, *a spider. The genome is of high quality, being chromosome-scaffolded, manually curated, and including the mitochondrion. The datasets are publicly accessible, as described in the manuscript. There are no errors or mistakes, and the method description is clear.

Are sufficient details of methods and materials provided to allow replication by others?

Yes

Is the rationale for creating the dataset(s) clearly described?

Yes

Are the datasets clearly presented in a useable and accessible format?

Yes

Are the protocols appropriate and is the work technically sound?

Yes

Reviewer Expertise:

bioinformatics, genome annotation

I confirm that I have read this submission and believe that I have an appropriate level of expertise to confirm that it is of an acceptable scientific standard.

# Reviewer report: Reviewer response for version 1

**DOI:** 10.21956/wellcomeopenres.24059.r94611

**Published:** 2024-08-23

**Authors:** Katharina Hoff

**Affiliations:** 1 University of Greifswald, Greifswald, Germany

The authors report the availability of the genome sequence of *T. montana*, a spider. The genome is of high quality (chromosome scaffolded, manually curated, mitochondrion also present). The data sets are publicly accessible as described in the manuscript.

 I have rarely seen such a flawless manuscript for peer review. There are errors, no mistakes, the methods description is clear.

 The only suggestion that I have is to add the structural annotation of protein coding genes, immediately. With **ERX11043128 ,** a paired end Illumina mRNA-Seq library exists. After repeat masking with Red or RepeatModeler2/RepeatMasker, you could easily apply BRAKER3 with the Arthropoda OrthoDB partition and that library to obtain the structural annotation of protein coding genes. 

 I am fully aware that it was not the authors' plan to do it at this point in time. Possibly EBI or NCBI have promised to annotate the genome, later. If that is the case, please ignore my suggestion. It just worries me that if neither EBI nor NCBI have promised to perform annotation, it is always difficult for third parties to add the annotation to the INDSC database on their own. Annotated genomes are more valuable than un-annotated genomes.

Are sufficient details of methods and materials provided to allow replication by others?

Yes

Is the rationale for creating the dataset(s) clearly described?

Yes

Are the datasets clearly presented in a useable and accessible format?

Yes

Are the protocols appropriate and is the work technically sound?

Yes

Reviewer Expertise:

bioinformatics, genome annotation, spiders

I confirm that I have read this submission and believe that I have an appropriate level of expertise to confirm that it is of an acceptable scientific standard.
